# Menopausal experiences of women of Chinese ethnicity: A meta-ethnography

**DOI:** 10.1371/journal.pone.0289322

**Published:** 2023-09-13

**Authors:** Md Ruhul Kabir, Kara Chan

**Affiliations:** 1 Department of Communication Studies, School of Communication and Film, Hong Kong Baptist University, Hong Kong, Hong Kong; 2 Department of Food Technology & Nutrition Science, Noakhali Science & Technology University, Noakhali, Bangladesh; Sapienza University of Rome, ITALY

## Abstract

**Objective:**

Menopause and the changes it brings to a woman’s life necessitate a comprehensive approach to face and experience the transition. This paper aims at synthesizing results from qualitative studies of menopausal experiences among Chinese and other women of similar ethnicity and culture.

**Design and method:**

A comprehensive search strategy of multiple databases along with bibliographic hand searches was employed to identify qualitative studies published in English peer-reviewed journals between 2008 and 2022 focused on the menopausal experiences (peri and post-menopause) of Chinese and other women of similar ethnic backgrounds. Twelve studies met the inclusion criteria. The final sample consisted of 238 women aged between 40 to 60 years who had experienced menopausal symptoms. This qualitative systematic literature review adopted Noblit and Hare’s seven-stage theoretical meta-ethnographic approach to construct an inductive and interpretive form of synthesis and subsequent analysis.

**Syntheses of findings:**

The synthesis of primary data identified four key concepts that entitle women’s menopausal experiences: being menopausal, ramifications on well-being, family and social support around menopause, and healthcare throughout menopause. The subsequent second-order interpretation revealed that women accepted the inevitability of the natural aging process in the decline of sexual drive, reinvented relationships with partners, and expressed the significance of a supportive environment in order to successfully navigate the transition. Third-order interpretations sought to establish a link between physiological complications, loss of femininity, patriarchal-dominated societal norms, and a support system that spans the entire menopause experience. Healthcare’s contribution has also been deemed to be insufficient due to a lack of information and empathy from health experts. Negligence or reluctance to seek healthcare and skepticism toward hormone replacement therapy (HRT) had also been a source of concern, as they have had the potential to exacerbate medical difficulties and emotional turmoil.

**Conclusions and implications for practice:**

A comprehensive approach that considers women’s physiological and psychological well-being and major attempts to change cultural beliefs and norms about women’s sexual health may be effective in aiding menopausal women during their transition. Additionally, appropriate guidelines and management should be in place to enable women to address menopause difficulties effectively with the assistance of healthcare experts and the support of their families and community.

## Introduction

Menopause is a natural degenerative process, a gradual rather than abrupt transition that marks the end of a woman’s reproductive potential. It is associated with aging, significant hormonal changes, and loss of fertility [1, 2]. However, women must have amenorrhea (absence of menstruation) for 12 months before being categorized as menopausal [3]. The early and late menopausal transitions, as well as the first year of early post-menopause, are all part of perimenopause. It usually starts in a woman’s late forties to early fifties and lasts about 15 years [1]. The years following menopause, whether natural (spontaneous) or premature, are referred to as post-menopause. Menopause usually begins between the ages of 50 and 51 [4] for Chinese women, with considerable variation among women living in different countries and regions [5]. Due to estrogen deprivation, women may have endocrine system imbalance and accompanying symptoms or disorders during this time. The loss of ovarian hormonal action during menopause, particularly the reduced amount of estrogen, can cause vasomotor, emotional, physiological, and atrophic alterations in estrogen-dependent tissues, contributing to the menopausal (climacteric) syndrome. The resulting changes can have a significant impact on women’s quality of life [6] and can even lead to several serious physical illnesses and mental stress [1, 7].

As menopause is sometimes linked to the onset of a wide range of symptoms, women frequently have preconceived views about it due to cultural stereotypes. There is a wide range of menopausal symptoms, as well as the connotations attached to this condition, in different countries and cultures [7]. Women going through menopause have no way of knowing if their experiences fall within the "normal" range. Menopausal symptoms such as hot flashes, heavy sweating, insomnia, mood swings, vaginal dryness, and decreased libido have all been reported, including affected interpersonal and social lives [7]. It implies that women’s bodies not only go through the menopausal transition but also their social and cultural identities. As a result of these modifications and adjustments, they are more susceptible to physical and psychological health issues. The formation of a conventional image of the menopausal woman as bad-tempered, frequently unpleasant, and beset by symptomatic difficulties has been reported frequently [8]. Negative social views regarding women going through menopause can have an impact on how menopausal symptoms are felt. Women’s opinions toward menopause symptoms are also influenced by how they experience menopause. Different cultural milieus contribute to various lifestyle behaviors, and perceptions of aging and women’s social standings affect how menopause is perceived [9, 10].

Additionally, as more women reach menopause, the majority would spend a significant amount of time in the post-menopausal years. This stage of life coincides with considerable changes in women’s lives, necessitating changes in a variety of areas. Family and marital relationships, sexual life, employment, and healthcare decision-making are all critical areas of adaptation that necessitate open communication with family members, partners, and coworkers [9, 11, 12]. There is a knowledge gap regarding strategies to enhance women’s quality of life during menopause and how to integrate all of these facets of life together. It is important to taking into account women’s sociocultural backgrounds, as well as their own subjective and personal perceptions. It also includes their perception of healthcare and how health providers contributed to the transition. To provide women with the greatest advice and treatment during this time by health providers, it is vital to understand the components that influence menopause [13]. This study also looks at how women perceived the role of healthcare and the contribution of health practitioners to the menopausal transition.

This meta-ethnographic study limits the scope of the menopausal experience to Chinese and other Asian women of similar ethnic origin (Taiwanese, Chinese Singaporean, Vietnamese, and women of similar backgrounds). Women who were going through or have gone through menopause are included in the study, with a particular emphasis on the social and cultural components of the transition. Because the meaning, attitudes, and behaviors related to menopause may vary depending on sociocultural factors [14]. Jin, Tao [15] argue that Asian midlife women, notably Chinese, have lower rates of physical and psychological symptoms and more favorable attitudes regarding menopause than other ethnic groups. However, the majority of the research on menopause uses clinically defined symptoms rather than women’s actual menopausal experiences [15]. Furthermore, there are some inconsistencies in the findings, with women reporting less positive attitudes toward menopause if they are less acculturated [16]. Asian women have reported invisible boundaries of cultural beliefs, values, and practices surrounding menopause that have been passed down from generation to generation. The nature of acceptance of these issues was marked by the gender roles in families and societies, as well as the conservative cultural characteristics that push women to be tolerant and emotionally stable around sexual health issues, including menopausal transition [12]. This study focuses on the physical, emotional, social, and cultural elements of menopause and how women have responded to it in traditional and patriarchal settings. Additionally, the evidence synthesis elucidates treatment alternatives, associated conundrums, and the degree of adjustment required to comprehend and embrace the transition, as well as embark on a new life journey.

Furthermore, this study is important in the sense that an increasing number of women in the Asia-Pacific region are approaching aging as a result of declining fertility and increased life expectancy. For instance, France took 115 years to transition from an aging to an aged society, whereas China will take 25 years, leaving them little time and opportunity to respond [17]. With an aging population, ensuring their health and well-being, particularly menopause-related health concerns, presents significant challenges that are exacerbated by sociocultural stigma. One study conducted in China revealed that while the majority of women were aware of menopause and associated symptoms, only a small percentage were mindful of hormone replacement therapy (HRT) and sought medical attention [11]. This qualitative synthesis of existing literature, therefore, aims to extract menopause-related knowledge and attitude as well as treatment and management concerns associated with it.

## Methods

The study used Noblit and Hare’s (1988) meta-ethnographic approach, which synthesizes previous qualitative research on a similar issue through a systematic comparison. This strategy extends beyond individual studies to elucidate the commonalities between them. It condenses the relevant literature while retaining its meaning through the use of essential metaphors (ideas, concepts, themes, perspectives, etc.) and organizers. This meta-ethnography does not try to summarize the entire corpus of information or make statistical inferences but concentrates on conceptual insight. The analogies uncovered in these translations serve as the meta-ethnographic synthesis’s structure [18] (S1 Fig). The Enhancing transparency in reporting the synthesis of qualitative research (ENTREQ) guidelines are used for reporting the synthesis of qualitative health research [19].

### Inclusion and exclusion criteria

The study included primary and original qualitative studies published in English in peer-reviewed journals between January 2008 and February 2022 that examined menopausal experience among women of Chinese or comparable ethnic origin. It includes women who are experiencing menopause and who have experienced menopause already but are already in the post-menopausal period. Menopause treatment studies, behavioral modification based on symptoms, and sociocultural factors of menopause based on those specific ethnic groups were also considered, regardless of the study’s location. Studies that included women who had undergone induced menopause (either as a result of surgical removal of the ovaries or as a result of iatrogenic ovarian function ablation) were excluded. Studies that focused exclusively on quantitative analysis of menopausal symptoms or a similar domain were excluded. However, mixed-method studies that included qualitative synthesis were included since they contained qualitative input that could add value to this study. A study that adopted a qualitative method of data collection but did not adopt a qualitative method of analysis was also excluded. We omitted reviews of any kind, whether systematic or meta-analysis/synthesis, commentary, brief reports, letters to editors, or theses, as well as government/organizational reports. Furthermore, the reference lists of the included studies and additional related studies were hand searched and reviewed to identify more primary qualitative research publications. We examined titles, abstracts, and full texts to eliminate papers that did not fulfill the eligibility requirements we established.

### Search strategy and data source

**Fig 1:**
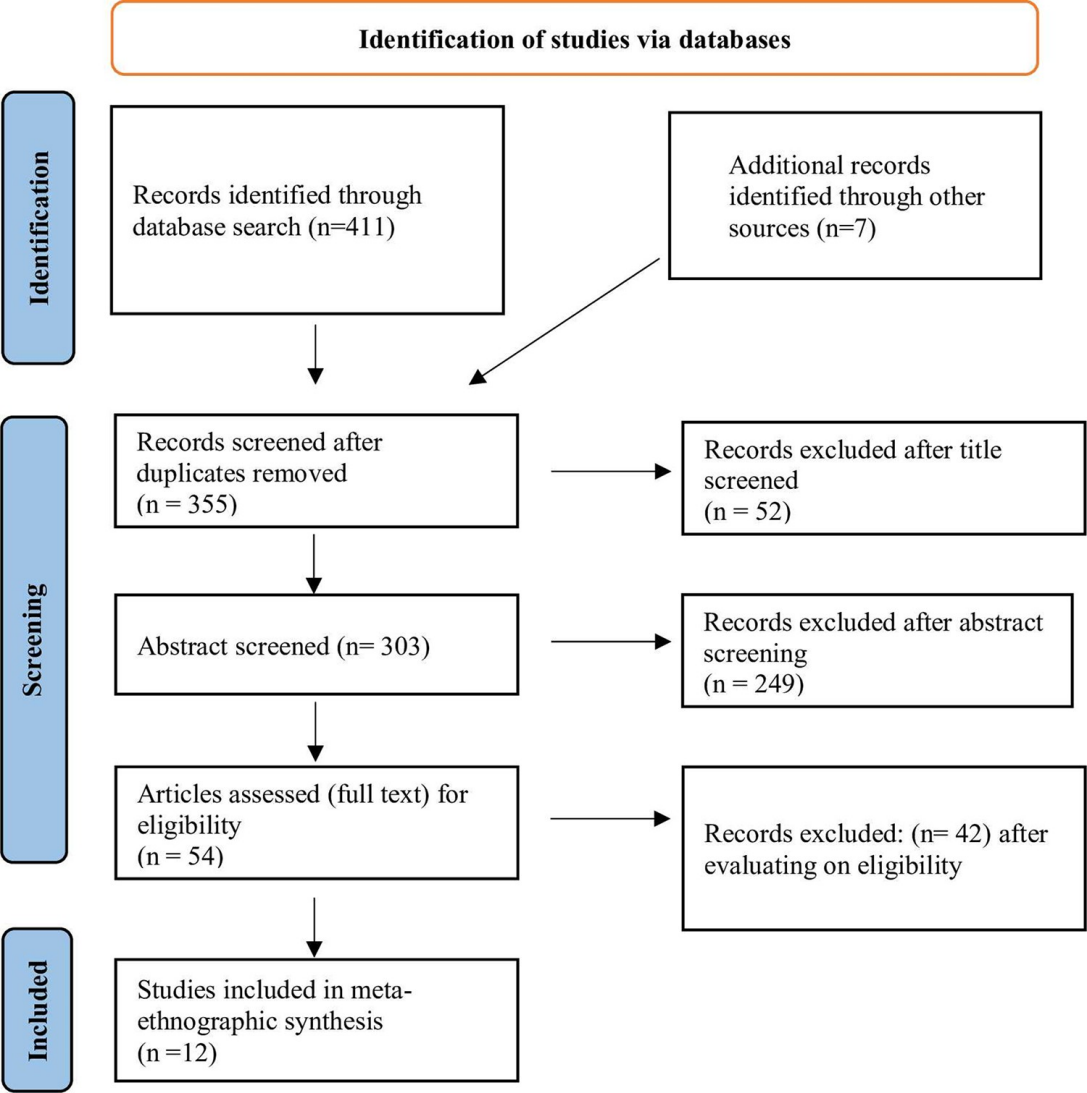
Flowchart of the study selection process.

We systematically searched electronic resources such as Google Scholar, PubMed, Ovid MEDLINE, CINAHL, EBSCO Host, and ProQuest Central (APA PsycINFO, Arts, and Humanities Database, Nursing & Allied Health Database, Psychology Database, Public Health Database, Social Science Database) for studies published between January 2008 and February 2022 with the help of an expert librarian. In addition to these databases, the Journal of Health Communication and Menopause was examined for potential matches. The time frame was chosen to provide the most up-to-date synthesis and analysis of findings, which are reflective of participants’ contemporary viewpoints. The study adopted a comprehensive search strategy to seek available studies that matches the inclusion criteria. Subject headings (and, where applicable, MeSH headings) and specific keywords were utilized in search categories that were customized for each database. Menopause or menopausal women; Experience and treatment; Chinese and other ethnic women; Qualitative study design filter are among the keywords for the search strategy. To discover and retrieve relevant articles, multiple subject headings and keywords were constructed from these concepts. Searches were conducted using terms such as “Menopause”, “Menopausal”, “Perimenopausal”, “Postmenopausal”, “Climacteric” “Experience”, “Expectations” “Needs”, “Concerns”, “Practices”, “Treatment”, “Relief”, “Chinese”, “Cantonese”, “Taiwanese”, “Malaysian”, “Singaporean”, “Qualitative”, “Grounded theory”, “Phenomenology”, “Ethnography”, “Interview”, “Content”, ‘Thematic”, “Discourse”, “Narrative”, etc. (these terms and texts words were combined interchangeably where applicable) (S1 Table). The bibliography of each relevant article was also evaluated to find related papers that might have been missed by database searches. The results from all searches were managed using EndNote software 20. The selection process of the included studies is illustrated in Fig 1.

### Study selection result

This meta-ethnography study included 12 studies, all of these studies are journal articles conducted among Chinese, Taiwanese, Chinese Singaporean, Vietnamese, and women of similar backgrounds (S1 Fig).

Selected articles were derived from the field of Nursing, Medicine, Public Health, Psychology, and Sociology. The final sample consisted of 238 women aged between 40 to 60 years who have experienced/ are experiencing menopausal symptoms.

### Qualitative appraisal

The Joanna Briggs Institute’s (JBI-QARI) critical appraisal checklist for interpretive and critical research (often referred to as a checklist for qualitative research) was used to evaluate the selected studies’ methodological quality. The JBI QARI critical appraisal tool employs 10 questions to examine and determine the extent to which a study’s design, methodology, and analysis addressed the likelihood of bias. Additionally, the selected studies were classified into high (8–10), medium (5–7), and low (<5) categories, a grading system established by one study [20] to comprehend the study’s respective contributions to the synthesis. This qualitative assessment is not intended to invalidate research with relatively minor methodological flaws or lack of rigor but rather to emphasize their contribution to the development of insightful concepts. This tool was selected because it is most compatible with our chosen storytelling strategy. Following confirmation of a fair agreement on the quality score between two authors on half of the chosen articles (Cohen’s Kappa: 0.615, p < .035), one author performed the remaining quality appraisals. The inter-rater reliability of qualitative appraisal methods is typically low, and there is little agreement on what constitutes an excellent or good enough qualitative study to be included in the final synthesis [21]. However, because these checklists are important for giving a subject for discussion, we have opted to evaluate them anyway (Table 1).

**Table 1:** Assessment of methodological quality of included studies.

Citations	Is there congruity between the stated philosophical perspective and the research methodology	Is there congruity between the research methodology and the research question or objectives?	Is there congruity between the research methodology and the methods used to collect data?	Is there congruity between the research methodology and the representation and analysis of data?	Is there congruity between the research methodology and the interpretation of results?	Is there a statement locating the researcher culturally or theoretically?	Is the influence of the researcher on the research, and vice- versa, addressed?	Are participants, and their voices, adequately represented?	Is the research ethical according to current criteria or, for recent studies, and is there evidence of ethical approval by an appropriate body?	Do the conclusions drawn in the research report flow from the analysis, or interpretation, of the data?	Quality appraisal
Im, Lee [12]	√	√	x	√	√	x	√	√	√	√	High
Lee, Lee [22]	√	√	√	√	√	√	√	x	√	√	High
Lim and Mackey [23]	√	√	√	√	√	x	x	x	√	√	Medium
Ling, Wong [24]	√	x	√	√	√	x	x	√	√	√	Medium
Mackey, Teo [25]	√	√	√	√	√	x	x	√	√	√	High
Mohamad Ishak, Jamani [26]	√	√	√	√	√	x	x	x	√	√	Medium
Ong, Chua [27]	√	√	√	√	√	x	x	√	√	√	High
Shea [28]	√	√	√	√	√	√	√	x	x	√	High
Stanzel, Hammarberg [29]	√	√	√	√	√	x	x	√	√	√	High
Vidayanti and Retnaningsih [30]	x	√	√	√	√	x	x	x	√	√	Medium
Wong, Huang [31]	√	√	√	√	√	x	x	√	√	√	High
Yang, Kenney [32]	√	√	√	√	√	x	x	√	√	√	High

Evaluation criteria: High: 8 or higher; Medium: 5–7; Low: <5; Based on Yes, No and Unclear evaluation of the 10-rating scale; √: Yes, x: No/unclear

### Data extraction, synthesis, and analysis

Data extraction and synthesis were carried out using Noblit and Hare’s (1988) seven-phase meta-ethnographic technique, refined by Campbell et al. (2012), which provided a rigorous framework for obtaining substantive interpretations from a collection of qualitative studies. These steps can be thought of as an iterative process that begins with the formation of a research idea worthy of synthesis, then moves on to finding relevant studies that meet the inclusion criteria, and finally, extracting concepts and ideas from the studies that are included. The next step is to determine how studies are related and linked to one another and then to translate primary study themes into conceptual categories through comparison. The translated synthesis also requires establishing a model that helps make sense of the entire synthesis by uncovering hidden insights in particular studies and communicating the whole synthesis to a larger population.

Information on each study’s aim, sampling, and sample profile, methodology, excerpts of participants’ responses, themes, and summary of findings were extracted into tables (Tables 2 and 3). Studies were arranged in alphabetical order. Results of all the selected studies were organized using MAXQDA software 20 for identifying codes. The studies are not coded into pre-existing concepts, rather concepts are produced after analysis and careful comparisons of the findings of each study. Both authors assessed all stages of the meta-ethnography process and aided in the synthesis process’s cohesive execution. One author aided in the selection and quality assessment of the synthesized articles, while the other checked the entire process for inconsistencies. The synthesis, selection of concepts and categories, and creation of lines of argument were likewise carried out concurrently by one author, with the other author assessing and reviewing the interpretations for appropriateness and relevance. Any discrepancy or incongruity in the procedure was discussed and resolved to the satisfaction of both authors.

**Table 2:** Details of included studies.

Source paper (Author/Year) and study location	Study aim	Participants (Number/Age, Ethnicity) (N = 238)	Research design/Stated methodology	Sampling, data collection and analysis
Im, Lee [12]	A feminist method was used to investigate the menopausal symptom experiences of Asian American midlife women.	Thirteen (13) Asian American women (including Chinese, Pilipino, Korean, and others) who have experienced menopause. Location: USA	Qualitative online forum study. Online forums are a feasible alternative to traditional face-to-face focus groups, especially for hidden populations with stigmatized conditions. A feminist approach	Participants were recruited through internet communities/groups among midlife Americans (ICMWs) and internet communities/groups for Asian Americans (ICAAs). Data were analyzed through thematic analysis.
Lee, Lee [22]	To develop a theoretical framework based on the experiences of menopausal women who have discontinued HRT.	Nineteen (19) Taiwanese women who discontinued HRT aged 45 to 67 years recruited from menopausal supporting groups. Location: North Taiwan.	This qualitative study; grounded theory approach	Purposive sampling; face-to-face in-depth interviews; constant comparative method was applied to analyze data.
Lim and Mackey [23]	This study examines ethnic Chinese women’s menopause transition experiences in Singapore.	Fourteen (14) menopausal and post-menopausal Chinese Singaporean women aged between 40–60 years who spoke English and attending a tertiary clinic. Location: Singapore	Qualitative study, used a purposive sampling technique	Audio-taped interviews, thematic analysis.
Ling, Wong [24]	To investigate difficulties and issues unique to postmenopausal women’s sexual practices and interests.	Twenty-two (22) postmenopausal women aged 50 and above from a metropolitan urban city in China were recruited. Location: China	Qualitative study, participants were contacted randomly from a telephone directory of an ongoing research	Focus group discussion; Grounded theory approach and content analysis.
Mackey, Teo [25]	To investigate menopausal transition-related knowledge, attitudes, and practices	Fifty-eight (58): Chinese (19), Malay (20), and Indian (19) Singaporean women aged between 40–60 years. Location: Singapore	A descriptive, qualitative study used interpretive paradigm	Convenience sampling, Interview with semi-structured interview guide, used thematic analysis
Mohamad Ishak, Jamani [26]	The purpose of this study is to elicit and describe women’s perceptions and understandings of their menopausal experience.	Twenty (20) menopausal women aged between 49 and 61 years Location: Pahang, Malaysia.	Qualitative study, sample collected through advertising on social media	Purposive sampling, in-depth semi-structured interviews, and thematic analysis.
Ong, Chua [27]	To gain a better understanding of the experiences and needs of perimenopausal women who are undergoing climacteric symptoms	Twenty (20) perimenopausal Singaporean women (Chinese being the major ethnic group) attending menopause clinic and experiencing at least one climacteric symptom. Location: Singapore	Descriptive qualitative study	Purposive sampling, semi-structured face-to-face interviews were conducted; data were analyzed through thematic analysis technique.
Shea [28]	To explore Chinese women’s perceptions and experiences of menopause and midlife ageing in comparison to research conducted in Japan, Canada, and the United States	Twelve (12) midlife Beijing women between the ages of 40 and 65. Location: Beijing, China	Mixed method ethnographic study (participant observation and formal interviews) along with survey.	Open-ended interviews, Participant observation was included.
Stanzel, Hammarberg [29]	To investigate menopause-related health literacy, menopausal experience, and encounters with healthcare.	Twelve (12) Vietnamese-born women immigrant in Australia aged between 45 and 60 years. Location: Australia	Qualitative study	Snowball sampling; Semi-structured interview; thematic analysis.
Vidayanti and Retnaningsih [30]	The purpose of this study is to examine the sexual experience of postmenopausal women.	Twelve (12) post-menopausal women in the age of 50–60 years. Location: Yogyakarta, Indonesia.	Phenomenology	Snowball sampling; in-depth face-to-face semi-structured interview. Conventional content analysis.
Wong, Huang [31]	To explore the impact of menopause on sexual health	30 Chinese Cantonese women aged between 43 and 64 years. Among them three were pre-menopausal, eight were peri-menopausal, and 17 were post-menopausal. Location: Hong Kong	Mixed methods study: Qualitative study to supplement quantitative findings	Purposive sampling, In-depth interviews, using a semi-structured, open-ended discussion guide, employed thematic analysis technique.
Yang, Kenney [32]	To examine Taiwanese women’s perceptions on menopause’s effect on their sexual lives	Eighteen (18) peri or post-menopausal women attending medical clinics for gynecological examinations who spoke Taiwanese or Chinese. Five of them aged <50 years, nine around 50–54 years, and three were more than 55 years aged. Location: Taiwan	Qualitative study from a post positivistic perspective	Face-to-face interviews using open ended questions. Content analysis technique was used for data analysis via inductive approach.

**Table 3:** Major findings of the selected studies.

Publication details	Categories/Themes	Quotations from participants in primary studies	Summary of findings
Im, Lee [12]	1. Being conditioned (by their cultural beliefs, values, and attitudes) • Invisible boundaries in the culture 2. Becoming strong 3. Appreciating (No pregnancy, and getting rid of menopausal symptoms) 4. Without making a fuss 5. Quiet support (Support from family, friends, online support group, etc.)	"Even though we have embraced contemporary methods of living and enhanced our standard of living, our values and beliefs have remained constant." "However, after seeing how my mother went through menopause over her lifetime, it appears to me that I am culturally conditioned." "The increased public knowledge and conversation about the issue in the United States inspire women like us, who come from a different society, to be more candid about it. Knowledge in and of itself is empowering." "In eastern cultures such as China, people avoid discussing menopause with strangers or even doctors."	Participants used adjectives like traditional, conservative, and patriarchal to define their cultural features surrounding menopause. Due to gender norms, women had to prioritize family demands, putting menopausal symptoms at the bottom of the priority list. Menopause was considered to be a natural, unavoidable, and transitory stage in a woman’s life. Because of cultural taboos and beliefs, which were asserted as women’s marginalized experience, women did not discuss their symptoms much. The participants in this study reported receiving positive support from their family members.
Lee, Lee [22]	Relieving discomforts safely • Immediately discontinuing HRT-it would hurt body (health threat, weight gain) • Symptoms bothering again (recurring menopausal discomfort: hot flushes, sweating, insomnia, irritability) • Negative emotions (confusion, frustration, worry, depression) • Learning to let it go (joined peer group to share experiences) • Trying to use nonHRT or products (Food with natural estrogen, nutritional supplements, TCM, therapies, etc.)	"Taking hormones may increase the risk of breast cancer." "I stopped taking hormone and suffered from all of the symptoms again within three months." "I’ve never felt this this before. I’m not interested in conversing with others." "I felt lethargic, fatigued, and overheated all of a sudden after stopping hormone. What’s the matter with me? I couldn’t get a good night’s sleep. "When you’re alone, you think too much. When you’re with other people, you’re more likely to notice things."	The study’s highlight was "choosing safe therapies and learning to let go," in which all participants attempted to identify different strategies to safely manage menopausal symptoms that were upsetting them. Most women stopped taking HRT for a variety of reasons, including health concerns and weight gain. Women were encouraged to try less expensive non-HRT therapies or other products, such as supplements or TCM, to alleviate their symptoms, which included hot flushes, insomnia, severe sweating, and other issues. Due to their frequent menopausal discomforts, women become depressed, confused, and wary. Women were seen adjusting their attitudes and thinking in peer support groups in order to learn to let go and control their frustration and pain.
Lim and Mackey [23]	1. Experiencing symptoms • Abnormal bleeding • Changing sex lives • Emotional changes (losing temper) 2. Experience of managing symptoms during menopause transition • Seeking medical help • Opted to TCM to manage bleeding problems or build up immune system as complementary with regular medication. • Support from family, especially from husband • Seeking information from various sources (friends, colleagues, social networks, books, magazines, etc.)	"It’s like if you sleep halfway and then feel extremely hot. Then you’ll. . . suddenly awaken, and then. . ." " I was exhausted and lacked sexual desire. . ." " I experienced hot flushes and cold flushes, and I was lethargic. . . I am unable to stand for two hours while working." "At times, you will simply lose your temper." "Menopause is a natural part of the aging process, and your life continues normally." "I took the hormone tablet for a few days and chose not to continue because I was unsure whether it would have any adverse effects." " I made an appointment with a Chinese physician. I used traditional Chinese medicine, but I continued to take my western medications." "Now I perform a variety of exercises. For instance, I practice yoga, taichi, and other forms of exercise in order to maintain my fitness. Yes, my hubby encouraged me to do so." “I did some research to see what other women were going through and things like these.”	Participants reported experiencing symptoms frequently linked with menopause, such as bleeding, hot flushes, and mood changes, yet the majority of them did not view this transition as troublesome, but rather as a natural part of life. Participants expressed a strong desire to adopt western medicine; symptom relief was cited as a reason for initiating HT. Support from family members, particularly the husband, was identified as a critical feature of the menopause transition. Obtaining information and exchanging experiences with friends was also emphasized.
Ling, Wong [24]	1. Perceived gender differences in sexual needs • Perceived discrepancies in sexual desire. 2. The role of marital relationship • Quality of marital relationship affects desire for love, affection, and sex • Vulnerable to marital issues and infidelity of husbands 3. Effects of sexual harmony • Poor martial relationships and irreconcilable differences. • Partners health issues • Environmental limitations (crowded living space) 4. Effects of menopause on sexual desire • Reduced sexual desire due to significant hormonal and physiological changes • Annoyance and rapid mood swings due to the displease caused by hot flushes. • Feeling free of concerns regarding pregnancies 5. Effects of social value judgment • Seldom talked to peers or friends about sexual issues • Being subjected to tag as crude and vulgar or horny by others. • Did not mention the use of TCM or other alternative medicine in its management.	"I will not think about sex on purpose, but I will meet my husband’s sexual desires once every four days." "This was on the verge of resulting in a divorce. ’No sex, no love,’ he intended. . . He made insulting and profane remarks about everything. . ." "I lost all sexual interest during menopause. I had already lost interest when I reached the age of 40-something. . ." " I’m no longer concerned. . . I have a greater sense of tranquility." "I have no objection to discussing sexual health at this meeting, but I have no reason to approach a stranger on the street. . ."	Disparities in the intensity of sexual cravings frequently resulted in marital discord, with spouses engaging in extramarital affairs. For Chinese women, cultural reluctance and fear of stigma operated as a barrier to seeking medical care. The majority of informants showed reluctance to address sexual health difficulties out of worry of how others might perceive them in society; yet they discussed their personal experiences openly in the study context. Menopause, environmental limits, and communication difficulties between couples all acted as impediments to healthy sexual interactions.
Mackey, Teo [25]	1. Knowledge • What happens at menopause • Sources of information 2. Attitudes • Acceptance (part and parcel of life) • Fear (About effects of menopause) • Aging (Sign of getting old) • Sharing experience (mixed feeling) 3. Practices (actions or behaviors) • Self-control • Medications and supplements • Seeing the doctor (if necessary) • Religion and prayer • Diet and exercise	"Menopause means that I will no longer have menses for the remainder of my years as a woman." "Because you have pals who inform you on this and that, you are more or less well prepared. I believe it is because of my job colleagues; I learn about their experiences.” "I’m forced to accept it because that is the way every woman is. . .I’m not looking forward to it, but I’m forced to confront it. "I’m not interested in losing the men, as you know. I’ve seen my friends who are older than me go through it, and they’ve changed dramatically." "We are females. “They, the guys, desire offspring from the women. I still have menstruation, you know, and I am still capable of having a baby." "I’m terrified, because it’s our turn now to enter old age." “I prefer to keep things personal.” "Misery in company is preferable to solitude" "I make an effort to maintain a cheery demeanor, and nobody is aware of these changes, particularly the psychological ones." " On occasion, I take sleeping drugs when I am unable to sleep" "I am not on HRT. I declined it due of its association with cancer." "A buddy suggested I try Chinese medicine, but I’m not sure because I’m allergic to it." "Hmm, only if you’re certain it’s severe enough that I should contact a doctor." "As you know, I beg to God that "Please, let me handle this." "I consume a great deal of fruit. “I exercise solely to lose weight."	Participants viewed menopause as a natural stage of life that must be endured. However, they perceived it as the end of youth, a sign that they were no longer attractive, and a symptom of aging. Frightened attitudes were prevalent among them, which implies a pervasive negative stereotype. There appears to have been a lack of comprehension about menopause and aging, which impacted symptom management and the use of HRT and other complementary and alternative remedies. Participants demonstrated reluctance to take medication or seek physicians for management, unless it’s severe to handle. They did not appear to seek information from health professionals.
Mohamad Ishak, Jamani [26]	1. Perception of menopause • Both negative and positive elements were found • Time for religious commitment 2. Biopsychosocial changes • Experiencing physical, psychological, and emotional changes. 3. Help seeking behaviors • Didn’t seek professional help • Lacking knowledge in seeking treatment	"Since menopause, my general health has deteriorated." "We do not need to be concerned about pregnancy during intercourse." "We were unable to pray or fast during menstruation. Now we can accomplish that." "Emotionally, I do feel bad. Previously, I was not overly emotional and was capable of controlling it. However, I’ve discovered that I’ve evolved." "When anything annoys me, I remain silent for a long. By doing so, I am able to comfort myself." "I was not compelled to seek treatment." "I used a variety of pills to enhance my hormone levels and to improve the appearance of my skin." "I have no idea about menopause treatment or health services. I had no idea menopause could be treated."	Menopause was thought of as an aging process that caused health problems and made women look and feel old. Participants appeared to accept menopause as a natural phase of life includes overcoming menstrual health issues and conceiving. Some women saw menopause as a chance to devote more time to their religion. Due to a lack of understanding, none of the participants sought medical therapy for their menstrual difficulties, preferring instead alternative treatments, vitamins, and herbal drugs.
Ong, Chua [27]	1. Uncertainty and misconception • Doubts over symptoms, management, treatment and health consequences. 2. Changes in the body • Physical symptoms (hot flushes, headache, fatigue, exhaustion, insomnia etc.) • Sexual experiences (Lower libido, vaginal dryness, painful intercourse) • Psychological symptoms (Mood swings, irritability, loss of temper) 3. Mixed feelings • Giving back to the younger generation (Innate responsibility to increase awareness) • Sense of ownership (sense of pride) • Feeling of being lost • Loss of womanhood • Aging and death 4. Social support • Sources of support (Family members, friends, online information) • Support for working women (Singapore’s busy lifestyle) 5. Wish list of women • Availability of information • Understanding and compassion • Empathetic healthcare professionals	"They (family and friends) advise against using hormone tablets. Because it is carcinogenic to the ovaries." "My mum suffered from these symptoms and succumbed to cancer. I’m having so many issues. . . I’m afraid that I, too, may succumb to cancer." " If you don’t sleep, you’re constantly exhausted. . ." "Now that I’m older, I have less sexual urge. . ." "I’m extremely upset." "When I attempt to share my experiences with them, they are appreciative." "I am aware that all women must go through this. Mentally, I did prepare myself." "I come from an age and a faith that do not discuss these subjects (menopause). . . ." "It’s dreadful (cries), I’ve lost my femininity. I feel as like I should tell my husband, "Go get another wife, since I am incapable of fulfilling my role as a wife." "Although my mother is an obstetrician, she never educated me. . ." " I seek knowledge online, but I am cautious of bogus sources." "Simply show us how to locate information, especially how to maintain a high quality of life during menopause." "I desire assistance at home." "Health personnel can be more careful and delicate with us, as we require assistance."	Women were skeptical about the symptoms, management, health concerns, and treatment options. Physiological issues included hot flushes, headaches, exhaustion, and insomnia, while psychological issues included mood swings and irritability. The loss of womanhood and a lack of desire for sex were both acknowledged aloud with grief. Positive feelings included a sense of ownership and giving back (by sharing experiences) to younger generations. There was a strong need to know where to access beneficial information, as well as for family members and health professionals to take a more supporting role.
Shea [28]	• The end of menstruation • The late midlife transition (the transition from middle to old age) • Hot flashes and female midlife in China • Emotional symptoms (irritable and vulnerable)	"After my periods stopped, I felt nice and clean. Less vexation." "I was sweating profusely, as if it were raining, and I didn’t see a doctor about it. I felt absolutely no embarrassment." • The majority of Chinese women place less emphasis on hormones and preferred to control their bodies through other means, such as herbal remedies or food. Many women made no mention of hormonal changes other than the cessation of menstruation. • The author observed that irritability in midlife was highly elaborated culturally, although depression was not. It focused on the vulnerability to anger and outbursts, which should not be suppressed but properly handled. "The majority of people believe that middle-aged ladies are rather irritated, and you should avoid debating this type of person." • According to the author, venting anger as a kind of moderation while maintaining some control and reserving it for personal or informal circumstances may not be viewed as self-indulgent.	Menopause and midlife ageing were identified as concepts and lived experiences that vary according to language, cultural ideas, and practices. When menstruation ended, some women felt free. Additionally, it was considered as a period of possible risk to mental, physical, and social health that must be managed carefully. Women described hot flushes differently, in less numbers than western women, and many Chinese women did not observe, or report hormonal changes associated with them. There were overwhelmingly positive attitudes of menstruation, and Chinese women appeared to downplay the importance of hormones, preferring to regulate their body changes through herbal medicine, food, exercise, and mediation. However, emotional shifts may cause some women to feel melancholy or irritated, and there was a certain cultural shame attached to it, there were tendency of others to avoid confronting menopausal women due to their irate nature. Personality and the capacity for self-control in the face of stressful circumstances can help alleviate midlife restlessness.
Stanzel, Hammarberg [29]	1. Menopausal experiences- It’s natural, it’s normal 2. Influence of culture on the experience of menopause • Sought support from friends and family and used traditional therapies • Limited health services and inadequate health information in Vietnam • Lack of health promotion program 3. Barriers for menopause-related health literacy • Appraising and understanding health information on the internet was difficult 4. Barriers and facilitators of optimal healthcare • Menopause and its effects on women’s health were not adequately discussed by providers. • Language difficulty	“I feel like we don’t need to do anything, and just accept it. I already knew about it from my friends.” “I take vitamin and eat a lot of fruit and veggie.” “My doctor did not advise any medical intervention and said it’s normal.” “I didn’t use medication even if my doctor prescribed some.” “They do not talk about menopause, yeah. . . They don’t think that this is important” (about healthcare in Vietnam) “I think doctors have to welcome, has to ask, invite question, rather than just prescribing.” “Female doctor is preferable.”	Women perceived menopause as a natural phase of life that does not require specific healthcare; rarely seek any professional help; however, when they did, they expect health professionals to initiate discussion rather than hasten it. There were language difficulties since women were residing in another country and they found it difficult to process and appraise health information found online or written in English. Therefore, the most common way to find information was from family and friends.
Vidayanti and Retnaningsih [30]	1. Negative changes in sexual functions • Decreasing frequency of sexual intercourse due to reduced libido, vaginal dryness and for discomfort and fatigue. 2. Intimacy with the couple • Keeping harmony of the relationship 3. Coping with the symptoms in postmenopausal symptoms. • Adapt and accept the situation, making an effort to increase healthy foods consumption and physical activities.	"I’m too weary and sluggish to have sex with my spouse after menopause, and we’re not getting the satisfaction we used to. . ." "My spouse no longer touches me as frequently as he used to. However, he is always discussing the issue we encountered." "Because the changes associated with menopause are unavoidable, I attempt to accept this situation. I attempt to alleviate the soreness with morning workouts."	Most of the participants experienced negative changes in sexual function, declining frequency of sex due to vaginal dryness and dyspareunia. Having sexual intercourse have not remain a pleasing experience for them. Communication and romanticism were enhanced and deepened through respectful and intimate conversation with partners. Husbands’ respectful involvement was a significant aspect to improve intimacy. Most participants accepted the menopausal changes positively and tried to engage themselves to do positive things like exercise, eating healthy foods, etc. That way, women became physically and mentally prepared to confront changes.
Wong, Huang [31]	1. Impact of menopause on marital life • Physical changes have a negative impact on sexual health • Differences in sexual drive within couples • A shift in focus of one’s marriage during menopause. 2. Difficulties encountered in marital sexual life and suggestions of support. • Cultural taboos and a suggestion for discussion • Inability to communicate due to a lack of understanding, support, and respect, as well as suggestions to communicate. • Lack of comprehensive information and suggestions to provide information. • Financial barriers to seeking advice from health professionals	"It’s been a while; I’m not interested in doing it now" (sex). Perhaps it is dry (vaginal). . .and there is no thrill at the moment." "At times, I attempt to accommodate, to make self-sacrifices. He (spouse) believes I fabricate excuses." "Now that we are becoming older, let us avoid doing anything stupid. . . He is considerate of my wishes." "Yes, there were instances when I went to speak; perhaps I did not feel comfortable inquiring. We did not discuss sex. . .” "Instead of speaking with friends, I would prefer to speak with medical specialists because my privacy would be protected. "I’m hoping they’re more understanding (men/husbands). . ." "Information should be more focused on sexual life, as there is already a wealth of other information available. . .for example, recordings of hot flashes." "I desired to consult TCM physicians, but financial constraints precluded me…"	Menopause had a significant detrimental effect on women’s sexual lives. Participants reported vaginal dryness and a lack of sexual desire. Some expressed anxiety about sharing information with peers owing to the potential for privacy violation. Men (husbands) were insufficiently considerate. There were barriers to receiving information about it and the overbearing physical and emotional changes that accompany it. Appropriate knowledge, financial assistance, and family support were reported to be beneficial.
Yang, Kenney [32]	1. Changes in physical responses during sex: • Vaginal dryness and pain • Loss of interest in sex • Continuing and maintaining a routine sex life. 2. The acceptance and non-acceptance of the current situation • Lacking and unsatisfying sex life. 3. Sexual pressure related to marital role • Feeling apologetic for the inability to meet the husband’s sex requirements • Readjusting the relationship as a couple 3. Efforts to improve sexual interest or activity • Taking hormone drugs • Communication and adjusting sexual positions and watching adult films • Increasing exercise	"I haven’t had sex in over a year as a result of vaginal dryness." "We’ve been lubricating, which is why it doesn’t seem dry." "We have had sporadic sex as he (husband) is incapable of creating an environment." "My spouse believes I was being uncooperative. It was a lack of libido. . .I was humiliated. . ." "The emphasis of life shifts. . . “Because we cannot be sexual partners, it is OK for us to be life partners." "It benefits my body and provides some compensation for my hubby. . ." "I believe that the couple should talk”. "Exercise improves physical strength and extends the duration of sex. . ."	Physical changes associated with menopause frequently complicated sexual encounters; nevertheless, the absence of sex is not always the result of menopausal changes. Some women expressed regret to their husbands, while others sought relief through behavioral adjustments, the use of lubricants during intercourse, or hormonal therapy. Accepting the changes, some women attempt to redefine their connection with their husband.

## Synthesis of findings

**Fig 2:**
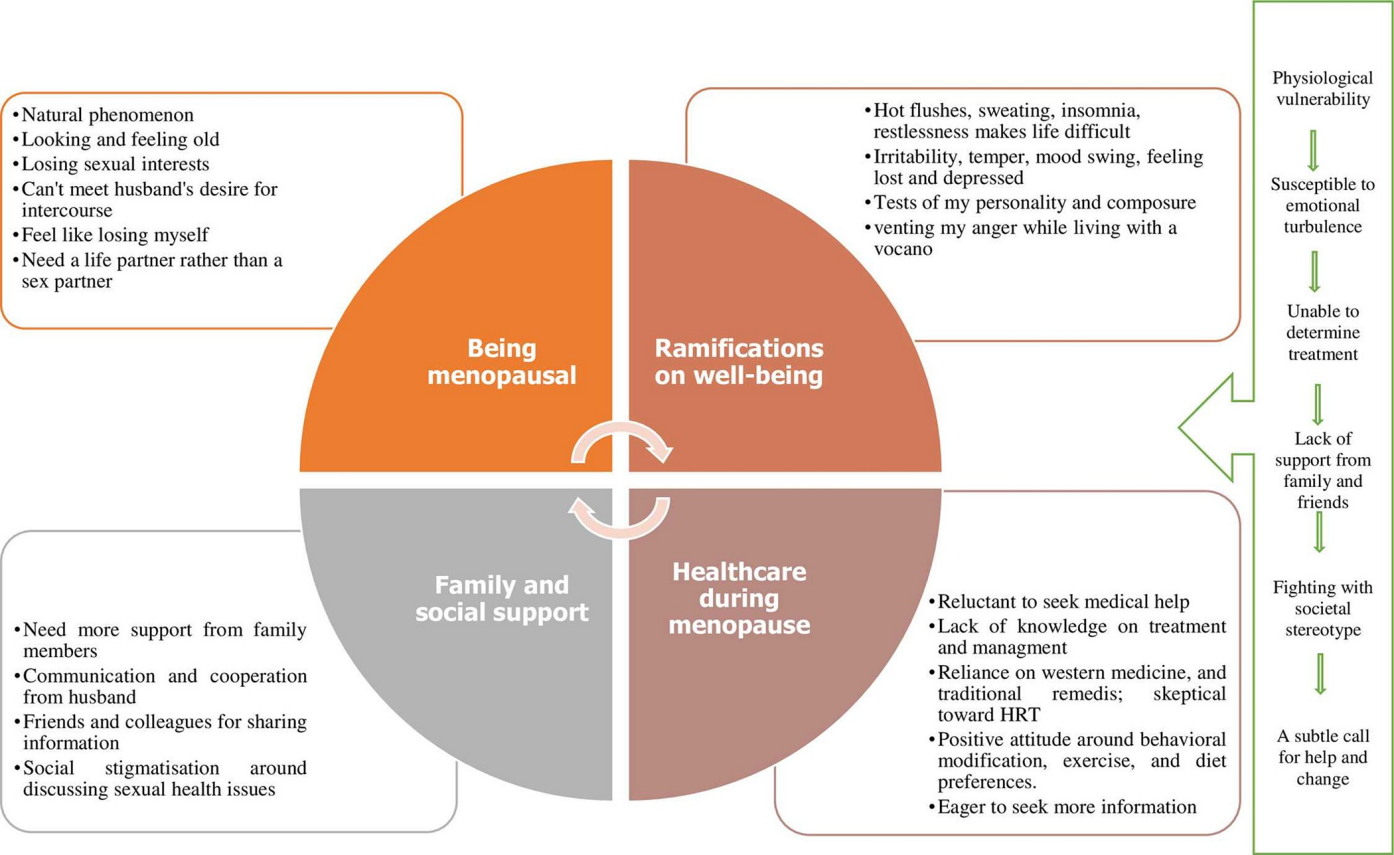
Line of argument: Menopausal experiences.

The qualitative synthesis of primary data identified four key concepts that entitle women’s menopausal experiences: being menopausal, ramifications on well-being, family and social support around menopause, and healthcare throughout menopause. Commonalities and differences in major concepts between studies were identified and compared (Table 4). Following that, key concepts were condensed (translated into one another) to produce second-order interpretations. Third-order interpretations were created based on reciprocal translations, which included comparing the findings and concepts from each study (lines-of argument) (Table 5). This line of argument resulted in a literary synthesis of menopausal women’s experiences that transcends individual studies and provides a comprehensive understanding of the issue under investigation (Fig 2).

**Table 4:** Identifying commonalities and differences between studies by comparing the major themes reported.

	Being menopausal			Ramifications on well-being		Family and social support		Healthcare during menopause	
Authors and year of publications	Menopause as a natural phenomenon	Aging process transition and rearguard myself	Decrease in sexual drive and effect on marital life	Adverse effect on physical and sexual health	Psychological turbulence and symptoms	Expectations and familial support (Specially from husband)	Societal enigma (Cultural beliefs, and taboos)	Menopause treatment, and management conundrum and concerns about HRT	Health information provision and cooperation from health providers
Im, Lee [12]	√	√				√	√		
Lee, Lee [22]		√			√		√	√	
Lim and Mackey [23]	√	√	√	√	√	√		√	√
Ling, Wong [24]		√	√		√		√		
Mackey, Teo [25]	√	√	√	√	√	√		√	√
Mohamad Ishak, Jamani [26]	√	√		√	√			√	
Ong, Chua [27]			√	√	√	√		√	√
Shea [28]	√	√		√	√		√	√	
Stanzel, Hammarberg [29]	√	√				√	√		√
Vidayanti and Retnaningsih [30]		√	√	√		√		√	
Wong, Huang [31]			√	√	√	√	√	√	√
Yang, Kenney [32]		√	√			√		√	
Number of studies with this theme	6	10	7	7	8	8	6	9	5

**Table 5:** Synthesis, comprising essential concepts and interpretations at the second and third order levels.

Key concepts and categories	Second-order interpretation	Third-order interpretation
Being Menopausal 1. Menopause is a natural phenomenon, and women have accepted it despite their reservations. 2. Transitioning through the aging process and rearguarding entails overcoming insecurities and diverting attention to useful endeavors. 3. Menopause decreased sexual drive, affected sexual life and dealt with spouse to reinvigorate marital relationship.	Women acknowledged the inevitability of the change, attempted to adapt to their new experience, reinvent their sexual drive and wants their husbands to be life partners. Some of them deplored to lose their sexual appeal, and look, and not being able to reproduce, while some found it refreshing and liberating. Despite the fact that their husbands were not always able to comprehend and collaborate, women were seen to divert their attention away from their relationships towards various activities that could make their lives more pleasurable or meaningful.	Women adjusted to menopause, embraced the transition, and discovered a new dimension to their sexual urge and subsequent sexual life with their partners despite some common setbacks. Diverse self-management strategies led their way to recovery. However, the linkage between the physiological and life transition put stress on psychological well-being. Menopausal women’s physical and emotional changes have been concerning while they lose their sexual prime. While they fight for their anxiety with their composure and personality, it also makes way to vent their anguish.
Ramifications on well-being 1. Adverse effects on physical and sexual health were evident. Hot flushes, vaginal dryness, heavy sweating, insomnia was among the common physical discomfort reported. 2. Psychological turbulence and losing myself illustrates the mental instability that menopause brings. Mood swing, temper, irritability, depression and not being control have been repeatedly mentioned.	Menopausal women go through physiological and psychological changes which had ramifications on their regular mundane life, sexual relations, and mental state. It resulted in lethargy, anxiousness, and an inability to manage one’s emotions, and rendered intercourse intolerable. The hormonal changes, symptoms that it brings, and resulting psychological state require more understanding to fathom whether physical complications lead to the depression or losing the womanhood that stressed up them much.	
Family and social standing around menopause 1. Woman expect support and understanding from the friends, and family members, especially from husband. Fear of privacy invasion, and difficulties in sharing problems were common concerns among women. Friends and colleagues, sometimes, came at a rescue where sharing was possible; however, it was never easy due to negative stereotype inside the household. 2. Cultural and religious beliefs, practices, and superstition around menopause	Understanding what a menopausal woman goes through may assist family members in overcoming the unfavorable stigma associated with it. Woman anticipated a positive atmosphere and sought collaboration and communication from her husband, who needed to reconsider their marital life as well. Concerns regarding how menopausal women’s changes are perceived by children and others were also pronounced. Bringing sexual health issues to the forefront and freely discussing them is viewed as culturally degrading and condescending.	Communication and cooperation from husband were the common aspects that have been reiterated from the menopausal women. Positive modifications and cultural acceptability are critical in dealing with traditional and patriarchal society views and practices.
Healthcare during menopause 1. Menopause management, treatment and behavioral modifications: There were evident lack of knowledge, and preparedness around menopause, and its symptoms. Concerns around its treatment were escalating, and women felt dubious of using HRT as treatment and its negative long term health effect. Rather they were comfortable using TCM, lubricants for sex, exercising or dietary modifications to re-energize their strength. 2. Limitations in health information were pronounced by some women and they sought more cooperation from health providers. Many women did not feel to seek professional help.	Menopause related health information seeking practice was questionable and women were reluctant to seek professional help unless absolutely necessary. Some women preferred western medicine, while some others opted for traditional medicines to manager their menopausal complications. They asked for relevant and accessible information and readjustment on health professionals views while managing menopause. There are significant room for improvement regarding how healthcare can facilitate the whole transition, from physiology- psychology- cultural perspective, not just manage it with medications.	Women who were skeptical about menopause treatment and management sought to supplement western medicine with traditional therapies. Concerns over healthcare’s contribution exacerbated their menopause-related healthcare-seeking behavior. The stigma around menopause management have been murky which signifies the stands of healthcare around it. The whole scenario implies a call for help and change that might facilitate the overall transition.

## Discussion

This meta-ethnography study of menopausal experiences among Chinese and other Asian women of similar ethnic origin was conducted following Noblit and Hare’s (1988) approach. It revealed crucial elements that shed insights for health educators and health professionals. The study depicted women’s experiences of aging, declining femininity, diminishing sexual desire, physiological issues brought on by hormonal changes, reinventing relationships with partners, and the struggle brought on by partner’s non-compliance. Anguish, frustration, and even emancipation from physiological binds were revealed in the synthesis. Women sought solace in seclusion as they prepared for a new journey. A therapeutic problem that extends from confusion about HRT to settling down to traditional therapies highlighted the menopause phenomena. This study emphasized the need for societal change around menopause and for healthcare to be more proactive in reaching out to women and promoting various coping strategies and resilience during menopause and sexual health.

Given that menopause permanently ends menstrual periods and results in a natural termination of fertility, it’s unsurprising that these changes resonated with most women in the study. Women’s transitions during the aging process and their attempts to adjust to those changes were re-iterated throughout the analytic process as women feared losing their womanhood. These hormonal alterations and metabolic changes occur in premenopausal women, making them susceptible to many disorders [33]. As a repercussion of all the changes, the third-order interpretation of this study regarding “being menopausal” revealed that women embraced the life transition mostly positively; however, not without some setbacks. These findings are resuscitated in another study in the UK where women expressed menopause as an “idiom of distress” [34].

Women seek alternative self-management skills to redeem themselves and adjust to their new life circumstances. This sense of "feminine identity loss" and subsequent reinventing and reclaiming of identity serves as a double-edged sword [35]. According to one study conducted among Sri Lankan women, menopause was the beginning of a new stage of life, not the end, and women discovered their own remedies through religious activities and increased community involvement [36]. However, the consequences for their emotional and psychological well-being were far-reaching, affecting their mood, causing insomnia and excessive sweating to the point where their families and society found it intolerable at times. After conducting an ethnographic study on Chinese women’s midlife ageing process, ethnographer Shea (2020) asserted that while emotional shifts might lead to feelings of irritation, releasing one’s anger in moderation can be beneficial if it is done privately [28]. Another study found that interventional tactics that address psychological distress linked with intense fatigue may help menopausal women cope with the midlife transition and enhance their mental health [37].

However, what regularly emerges in the women’s narratives is family and societal support, which may be tied to how menopause was perceived in society. Cultural taboos and superstitions were associated with it, as addressing and discussing sexual health issues, including menopause, was viewed as culturally degrading and condescending. Women desired increased cooperation and communication from family members, especially husbands and society at large. Acceptance and constructive change of the culture was crucial in dealing with menopause in the Chinese patriarchal and traditional society. Because attitudes and sociocultural views shape the setting in which women experience menopause, cultural influences have a significant impact on how women perceive and manage menopausal symptoms [9]. Research on immigrant South Asian women in Canada discovered a dearth of personal support for menopausal women from their families and communities, indicating the need for culturally appropriate community-based initiatives [38]. This emphasizes the necessity of culturally sensitive approaches to menopause, and this meta-synthesis presented a compelling metaphor beyond cultural stigmatization introduced by traditional patriarchal society. It reflects how women’s sexual health issues have been approached in a society dominated by male bravado. The line of argument attempted to connect the issues of physiological complications, psychological insecurities, and a lack of meaningful support from families and societies, as the level of social support may affect the menopausal transition [39].

For many participants, being in a marginalized physical and psychological state and sometimes not receiving enough support from others was also exacerbated by a lack of meaningful information and confusion about the treatment and management of menopause. Friends, colleagues, or peers occasionally stepped forward to assist women in navigating menopause. Women were perceived as being neglectful, hesitant, or embarrassed to seek medical care from a health professional until absolutely required. Women were unaware of who to turn to for help or where to look for it. This finding is consistent with one study, which said that two major obstacles for menopausal women were not knowing how to access information correctly and not being aware of credible sources of information [40]. The healthcare sector’s contribution and information provision were deemed insufficient, along with health providers’ motivation to provide sufficient information. Health professionals should proactively engage with women as many of them may not open up to express their concerns and prefer to keep their problems to themselves [41].

The meta-analysis also indicated that many women voiced concern about the long-term consequences of HRT, with friends and family advocating them not to use it. Treatment of menopause difficulties and dealing with menopause often involves the use of traditional therapies such as improved nutrition and exercise, lubricant usage, and organizing ideas to condense personality. Research into traditional remedies and women’s reliance on this component is something to keep an eye out for in the future. The influence of traditional therapies and women’s reliance on this is something to look for in future research. Information regarding hormonal therapy, the effect of physical exercise [42], influencing women to seek health information, and enabling support groups might be useful to improve the quality of life of menopausal women [36].

The present synthesis generated a line of argument model that encompasses most of the critical aspects of menopausal experiences as iterated in various studies, and the analysis concludes that in order to engage menopausal women in healthcare, it is critical that the community educates itself about it, accepts and discusses it, and facilitates the tumultuous transition of women who were already undergoing significant physical and psychological changes. Because it gets harder when women have to resist the societal juggernaut while already in a quandary or feeling lost. Menopause’s physiological, psychological, social, and healthcare aspects should be addressed concurrently and coherently to take a complete strategy to alleviate the pain, agony, and bewilderment associated with menopause.

### Strengths and limitations

This meta-ethnography study is not devoid of limitations. This study focused exclusively on the experiences of Chinese women or women of comparable ethnic origin in Asia. As a result, the study findings do not reflect all women from diverse sociocultural backgrounds. The study concentrated on menopausal experiences and excluded studies that lacked this component. Because methodological rigor was not used as exclusion criteria in this study, the synthesis and subsequent analysis cannot claim to be of the highest quality. However, the multidisciplinary perspective of the included studies (nursing, medicine, and sociology) is a significant strength, as it extends to translating and understanding the inner meanings of menopausal experience-related study findings. It offers the necessary richness beyond the specific focus of individual studies and encompasses the diversity of menopausal experiences that necessitates a thorough understanding. The study examined research findings that considered cultural and health factors, and health professionals and policymakers can use lines of argument to develop a comprehensive plan that views menopause not only as a health concern in need of direction but also as a societal issue requiring collective effort to overcome.

## Conclusions

This meta-ethnographic study demonstrated that Chinese and Asian women of related ethnic origins’ menopausal experiences are inextricably linked to their sociocultural stance, which spans from suffering in silence to rediscovering confidence to begin anew. This study’s synthesis and interpretations established that menopause is a complex phenomenon that encompasses numerous health and social facets. Menopausal women had a common sense of bewilderment, sadness, and uncertainty over menopause and its management, yet they accepted it as natural and unavoidable. They anticipate a more supportive atmosphere from their surroundings and aid from healthcare, whether in the form of information or proactive provider participation. Social conventions, taboos, and stigma have deteriorated to the point where the entire issue demands a shift in attitude, education, and positive health promotion addressing all aspects of sexual health, not only menopause. Appropriate scientific and culturally sensitive treatment and management plans integrating traditional remedies, diet, and exercise plans, as well as reasons and measures to take good mental health initiatives, should be a key emphasis for menopausal women. Disintegrated approaches may miss the mark by failing to evaluate all aspects of menopause, leaving some components ignored, unanswered, and unaccounted for.

## Supporting information

S1 Fig Stages of meta-ethnography synthesis. (DOCX)

S2 Fig Flowchart of selection process (PRISMA chart). (DOCX)

S1 Table Keyword search and concepts. (DOCX)
