# Supplementary material for: Menopausal experiences of women of Chinese ethnicity: A meta-ethnography
Source: PLoS One. 2023 Sep 13;18(9):e0289322. doi: 10.1371/journal.pone.0289322 (PMC10499211; doi:10.1371/journal.pone.0289322)
Supplement: S1 Fig — (DOCX) [file pone.0289322.s001.docx]

Title: Stages of meta-ethnography synthesis process according to Noblit and Hare (1988)

Research idea formulation

Initial extraction of findings and concepts:

Conceptual content of the individual studies is extracted

Finding relevant studies and deciding on inclusion criteria

Determining how studies are related to each other:

Identifying, describing, and comparing themes and categories (First- order- interpretation)

Sorting concepts and themes into conceptual categories/themes:

Translating studies into one another to achieve second-order-interpretation

Overall textual synthesis to develop a model: A lines-of-argument*:

A third order-interpretation involves reading the textual synthesis for each thematic group

Expressing the overall textual synthesis

Dissemination of the findings to hope policymakers may find it useful

Figure: Stages of meta-ethnography synthesis process according to Noblit and Hare (1988)

* Lines-of-argument synthesis entails first translating research into one another and then developing an interpretation that may aid in uncovering what was hidden in individual studies in order to highlight the overarching synthesis.
