# Supplementary material for: Menopausal experiences of women of Chinese ethnicity: A meta-ethnography
Source: PLoS One. 2023 Sep 13;18(9):e0289322. doi: 10.1371/journal.pone.0289322 (PMC10499211; doi:10.1371/journal.pone.0289322)
Supplement: S2 Fig — (DOCX) [file pone.0289322.s002.docx]

**Identification of studies via databases**

Records screened after duplicates removed

(n = 355)

Records excluded after title screened

(n = 52)

Additional records identified through other sources (n=7)

Records identified through database search (n=411)

**Identification**

**Screening**

Records excluded after abstract screening

(n = 249)

Abstract screened (n= 303)

Records excluded: (n= 42) after evaluating on eligibility

Articles assessed (full text) for eligibility

(n = 54)

**Included**

Studies included in meta-ethnographic synthesis

(n =12)

**Figure 1: Flowchart of selection process**
