# Supplementary material for: Menopausal experiences of women of Chinese ethnicity: A meta-ethnography
Source: PLoS One. 2023 Sep 13;18(9):e0289322. doi: 10.1371/journal.pone.0289322 (PMC10499211; doi:10.1371/journal.pone.0289322)
Supplement: S1 Table — (DOCX) [file pone.0289322.s003.docx]

**Keywords search and concepts**

| **Menopause** | **Experience** | **Qualitative study** | **Country/Ethnicity** |
| --- | --- | --- | --- |
| Menopause | Experience | Qualitative | China OR Chinese |
| Menopausal | Expectations | Grounded theory | Other Asian Countries (Taiwanese, Vietnamese, Malaysian, Macao, etc.) |
| Perimenopause | Sex life OR married life | Phenomenology |  |
| Perimenopausal | Hormone therapy (HRT) | Ethnography |  |
| Climacteric Symptoms | Culture OR  Societal | Interview OR Narrative OR Focus group |  |
|  |  | Content OR Thematic OR Comparative Discourse Analysis |  |

Sample search strategy in Ovid Medline

| Ovid MEDLINE(R)  1 Menopause, Premature/ or Menopause/ or Menopause.mp.  2 Menopause/ or Climacteric/ or Perimenopause/  3 1 or 2  4 Chinese.mp. or Asians/  5 3 and 4  6 Qualitative Research/ or Qualitative.mp.  7 Ethnography.mp. or Anthropology, Cultural/  8 Narrative.mp.  9 Phenomenology.mp.  10 Interviews as Topic/ or Grounded Theory/ or Qualitative Research/ or Grounded.mp.  11 Comparative.mp. or Comparative Study/  12 Discourse.mp.  13 Adaptation, Psychological/ or Content analysis.mp. or Qualitative Research/  14 Thematic.mp. or Qualitative Research/  15 6 or 7 or 8 or 9 or 10 or 11 or 12 or 13 or 14  16 5 and 15  17 Singapore.mp. or Singapore/  18 Malaysia.mp. or Malaysia/  19 Vietnam.mp. or Vietnam/  20 Taiwan.mp. or Taiwan/  21 Macao.mp. or Macau/  22 17 or 18 or 19 or 20 or 21  23 16 and 22 |
| --- |
